# The Effect of Sclerostin and Monoclonal Sclerostin Antibody Romosozumab on Osteogenesis and Osteoclastogenesis Mediated by Periodontal Ligament Fibroblasts

**DOI:** 10.3390/ijms24087574

**Published:** 2023-04-20

**Authors:** Karina E. Pigeaud, Melanie L. Rietveld, Aster F. Witvliet, Jolanda M. A. Hogervorst, Chen Zhang, Tim Forouzanfar, Nathalie Bravenboer, Ton Schoenmaker, Teun J. de Vries

**Affiliations:** 1 Department of Periodontology, Academic Centre for Dentistry Amsterdam (ACTA), University of Amsterdam and Vrije Universiteit, Gustav Mahlerlaan 3004, 1081 LA Amsterdam, The Netherlands; k.e.pigeaud@acta.nl (K.E.P.);; 2 Amsterdam University College, University of Amsterdam and Vrije Universiteit, Science Park 113, 1098 XG Amsterdam, The Netherlands; 3 Department of Oral Cell Biology, Academic Centre for Dentistry Amsterdam (ACTA), University of Amsterdam and Vrije Universiteit Amsterdam, Amsterdam Movement Sciences, 1081 LA Amsterdam, The Netherlands; 4 Department of Clinical Chemistry, Amsterdam University Medical Centers, Vrije Universiteit Amsterdam, 1081 HV Amsterdam, The Netherlands; 5 Oral Pathology and 3D Innovation Lab, Department of Oral and Maxillofacial Surgery, Amsterdam University Medical Center, Vrije Universiteit Amsterdam, Amsterdam Movement Sciences, 1081 LA Amsterdam, The Netherlands

**Keywords:** periodontal ligament fibroblast, osteogenesis, osteoclastogenesis, sclerostin, romosozumab, bone balance

## Abstract

Sclerostin is a bone formation inhibitor produced by osteocytes. Although sclerostin is mainly expressed in osteocytes, it was also reported in periodontal ligament (PDL) fibroblasts, which are cells that play a role in both osteogenesis and osteoclastogenesis. Here, we assess the role of sclerostin and its clinically used inhibitor, romosozumab, in both processes. For osteogenesis assays, human PDL fibroblasts were cultured under control or mineralizing conditions with increasing concentrations of sclerostin or romosozumab. For analyzing osteogenic capacity and alkaline phosphatase (ALP) activity, alizarin red staining for mineral deposition and qPCR of osteogenic markers were performed. Osteoclast formation was investigated in the presence of sclerostin or romosozumab and, in PDLs, in the presence of fibroblasts co-cultured with peripheral blood mononuclear cells (PBMCs). PDL-PBMC co-cultures stimulated with sclerostin did not affect osteoclast formation. In contrast, the addition of romosozumab slightly reduced the osteoclast formation in PDL-PBMC co-cultures at high concentrations. Neither sclerostin nor romosozumab affected the osteogenic capacity of PDL fibroblasts. qPCR analysis showed that the mineralization medium upregulated the relative expression of osteogenic markers, but this expression was barely affected when romosozumab was added to the cultures. In order to account for the limited effects of sclerostin or romosozumab, we finally compared the expression of SOST and its receptors LRP-4, -5, and -6 to the expression in osteocyte rich-bone. The expression of SOST, LRP-4, and LRP-5 was higher in osteocytes compared to in PDL cells. The limited interaction of sclerostin or romosozumab with PDL fibroblasts may relate to the primary biological function of the periodontal ligament: to primarily resist bone formation and bone degradation to the benefit of an intact ligament that is indented by every chew movement.

## 1. Introduction

Periodontitis is a frequently occurring inflammation-associated condition affecting the teeth, and, when untreated, it leads to the destruction of supporting tissues surrounding the teeth [1]. In the United States, among adults above 30 years of age, the prevalence of periodontitis is 47% [2]. This disease is characterized by a loss of connective tissue and a loss of the periodontal ligament (PDL) with a preponderance of alveolar bone resorption [1]. Periodontitis can manifest itself in tooth mobility, impaired masticatory ability, compromised esthetics and eventually tooth loss [1]. Periodontitis has also been associated with increased risk of chronic diseases, including atherosclerotic cardiovascular diseases and diabetes [3,4].

Different non-surgical and surgical therapies are used for the treatment of periodontitis. None of these treatments are satisfactory in terms of reconstructive and disease modifying capabilities due to limited predictability and efficacy [5,6]. In this context, the glycoprotein sclerostin, encoded by the SOST gene, has emerged as a new research topic for periodontitis treatment. Sclerostin plays an important role in bone remodeling as an inhibitor of bone formation. Sclerostin decreases bone formation via blocking the canonical Wnt-signaling pathway by binding with the Wnt co-receptors LRP 5/6 [7]. Co-receptor LRP4 can also bind to sclerostin and, thus, enhance the inhibition of bone formation [8]. Sclerostin is mainly expressed by osteocytes, cementocytes, and hypertrophic chondrocytes [9]. Next to these cells, sclerostin has also been identified in PDL fibroblasts [10,11,12,13].

The absence of sclerostin causes sclerosing bone disorders such as sclerosteosis or Van Buchem Disease. These diseases are characterized by generalized bone overgrowth due to an increased osteoblastic activity that is not counterbalanced by increased osteoclastic activity [9]. The recognition of the clinical consequences of sclerostin absence stimulated the development of sclerostin inhibitors as a potential therapy for bone disorders characterized by excessive bone resorption, such as osteoporosis [14]. As a result, romosozomab, a monoclonal antibody, was developed, which binds and inhibits sclerostin, leading to an increase in bone formation and a decrease in bone resorption [15]. In 2019, romosozumab received its first global approval by the Food and Drug Administration for treating osteoporosis in patients with a high risk of fracture [16], which was followed by its approval for use in the European Union [17]. 

In recent years, the monoclonal sclerostin antibody has also been investigated in pre-clinical studies in the dental field, for example, as a promising therapy in augmenting alveolar bone healing and stimulating bone regeneration in rats with a surgically generated bone defect [18]. Taut et al. concluded that the monoclonal sclerostin antibody stimulated bone regeneration in rats following experimental periodontitis [18]. Moreover, Chen et al. proved that a monoclonal sclerostin antibody treatment stimulated alveolar crest height and bone mass in ovariectomized rats with localized periodontitis [19]. Moreover, Liu et al. reported that sclerostin inhibition in rats with alveolar bone loss resulted in the additional stimulation of alveolar bone volume [20]. Similarly, Yao et al. evaluated the effect of sclerostin antibodies on osseous defects around teeth [21]. They showed that the systemic administration of sclerostin antibodies enhanced bone regeneration; however, locally applied sclerostin failed to improve bone repair [21]. 

Studies on sclerostin in dentistry have mainly focused on the effect of sclerostin and sclerostin blocking antibodies in the osteocytes of the alveolar bone. Although sclerostin is mainly produced by osteocytes, several studies suggest a role for PDL fibroblasts in sclerostin signaling. Jäger et al. discovered that increasing levels of SOST mRNA and protein were identified in PDL fibroblast cultures that were induced to form minerals [12]. In addition, different studies have demonstrated that PDL fibroblasts respond to mechanical force by increasing the expression of SOST, both at gene and protein levels [22,23]. 

Nevertheless, the regulatory mechanisms and effects of sclerostin and monoclonal sclerostin antibodies on biological parameters such as the osteogenesis and osteoclastogenesis of PDL fibroblasts are still unknown. These mechanisms and effects are potentially of value for the development of regenerative therapies in periodontitis patients. The purpose of this study is to explore the effect of sclerostin and a monoclonal sclerostin antibody, more specifically romosozumab, on osteoclastogenesis and osteogenesis in PDL fibroblast cultures. 

PDL fibroblast cultures have been widely studied in bone remodeling research in dentistry, as PDL fibroblasts play an important role in both osteogenesis and osteoclastogenesis [24]. PDL fibroblasts have the capacity to contribute both to mineralization, due to their high alkaline phosphatase (ALP) activity, and to osteoclast formation in co-cultures with peripheral blood mononuclear cells (PBMCs) [24,25]. The osteogenic capacity of PDL fibroblast cultures can be investigated by stimulating the cells with a mineralization medium consisting of ascorbic acid and β-glycerophosphate [12,26]. PDL fibroblasts, in a co-culture with PBMCs, can provide the signals that stimulate the monocytes within the PBMCs to differentiate into osteoclasts [27]. 

The hypothesis is that the addition of sclerostin to PDL fibroblasts results in decreased mineralization and osteoblast activity and the addition of a blocking monoclonal antibody against sclerostin will result in increased mineralization and osteoblast activity. This study further hypothesized that the addition of sclerostin to a co-culture of PDL fibroblasts and human PBMCs results in enhanced osteoclastogenesis, whereas the addition of a monoclonal sclerostin antibody, romosozumab, will lead to decreased osteoclastogenesis.

## 2. Results 

### 2.1. Osteoclastogenesis 

**Figure 1:**
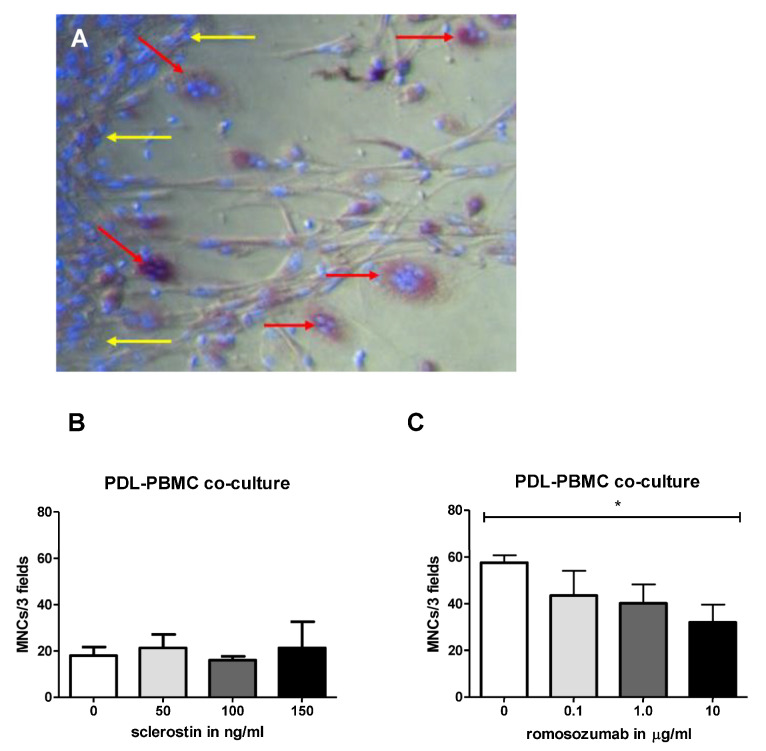
Periodontal ligament-associated osteoclast formation. (**A**) The retraction of PDL fibroblasts (yellow arrows) and the migration of PBMCs to the exposed plastic where multinucleated TRAcP-positive cells develop (red arrows). Micrograph was taken after 21 days of co-culture. Scale bar represents 100 µm. (**B**) Effect of increasing concentrations of sclerostin in a PDL-PBMC co-culture. The number of multinucleated cells formed in a PDL-PBMC co-culture in the presence of sclerostin (50–150 ng/mL). Osteoclast formation did not differ significantly between the conditions. (**C**) The number of multinucleated cells formed in a PDL-PBMC co-culture in the presence of romosozumab (0.1–10 µg/mL). The number of multinucleated cells was significantly reduced at 10 µg/mL romosozumab in comparison to the control group (**B**). Assays were performed using three (sclerostin) or five (romosozumab) different donors in duplicates, average results ± SD are shown. * *p* < 0.05. Sclerostin and romosozumab experiments were separate experiments with different PDL-donors and PBMC donor. Control values in both experiments differ, likely due to the different PBMC donor.

Osteoclasts, identified as multinucleated TRACP-positive cells, were formed during all of the osteoclastogenesis experiments. For the PBMC-PDL co-cultures, a specific sequence of osteoclast formation was noticed. Initially, the PDL fibroblasts and PBMCs adhered to each other and were evenly distributed over the well. Later, after 7–14 days, the PDL fibroblasts started to retract. Subsequently, multinucleated TRAcP-positive cells formed on the plastic where the PDL fibroblasts had retracted (Figure 1A). 

#### 2.1.1. The Addition of Romosozumab Slightly Influences the Osteoclastogenic Capacity of PDL-PBMC Co-Cultures

For osteoclastogenesis assays, PDL fibroblasts and PBMCs were co-cultured for 21 days with increasing concentrations of sclerostin (0, 50, 100, and 150 ng/mL) or romosozumab (0, 0.1, 1, and 10 µg/mL). To determine the number of osteoclasts and the number of nuclei per osteoclasts, TRAcP-positive cells with more than three nuclei were counted and categorized as follows: 3–5, 6–10, and >10 nuclei. Nearly all multinucleated cells belonged to the first group (3–5 nuclei); therefore, the three categories were merged. When the PDL-PBMC co-culture was stimulated with sclerostin, no significant difference in the number of TRAcP-positive multinucleated cells was detected (Figure 1B). Conversely, the addition of romosozumab appeared to be associated with a decreased number of TRAcP-positive multinucleated cells (Figure 1C). This result was only statistically significant for the 10 µg/mL romosozumab condition in comparison to the control group. 

#### 2.1.2. Sclerostin and Romosozumab Do Not Affect the Osteoclastic Capacity of High-Density Cell Cultures

To analyze the effect of sclerostin and romosozumab under conditions devoid of PDLs, the spontaneous osteoclastogenesis of PBMCs was assessed. PBMCs were cultured in quadruplicate per condition on plastic with increasing concentrations of sclerostin or romosozumab. After 21 days of culturing, osteoclasts were identified with TRAcP staining. Osteoclasts formed under all conditions. 

**Figure 2:**
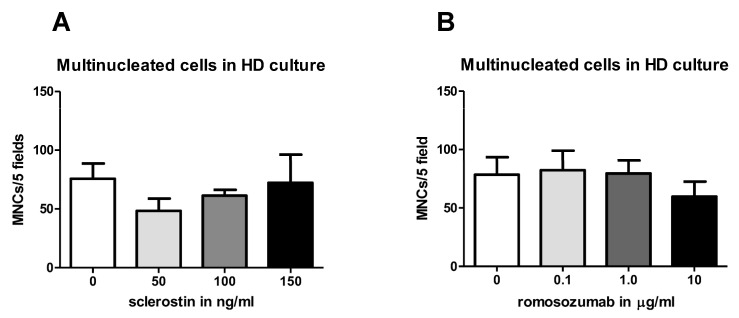
TRAcP-positive multinucleated cells in HD culture. (**A**,**B**) Effect of increasing concentrations of sclerostin and romosozumab in a PBMC high density (HD) culture. (**A**) The number of multinucleated cells formed in a PBMC HD culture in the presence of sclerostin (50–150 ng/mL). The number of multinucleated cells formed in a PBMC HD culture in the presence of romosozumab (0.1–10 µg/mL). No significant differences were observed. PBMCs HD culture experiments were performed in quadruplicate. Mean results ± SEM are presented. Multinucleated cells with more than 3 nuclei were counted and categorized: 3–5, 6–10, and >10 nuclei. Nearly all of the multinucleated cells had 3–5 nuclei; therefore, categories were merged in this figure.

The number of osteoclasts in the sclerostin group and the romosozumab group was comparable. When adding sclerostin, no differences in osteoclast formation were observed (Figure 2A). Furthermore, the addition of romosozumab did not affect osteoclast formation (Figure 2B). 

### 2.2. Osteogenesis

#### 2.2.1. Neither Sclerostin nor Romosozumab Affected the Osteogenic Capacity of PDL Fibroblasts

To establish the effect of sclerostin and romosozumab on osteogenesis, several experiments were performed. As a marker for bone formation, ALP activity was assessed and Alizarin red staining was performed to study mineral deposition by PDL fibroblasts. Finally, the gene expression of osteogenic markers was assessed in the presence of romosozumab. 

#### 2.2.2. Alkaline Phosphatase Activity and Cell Proliferation

**Figure 3:**
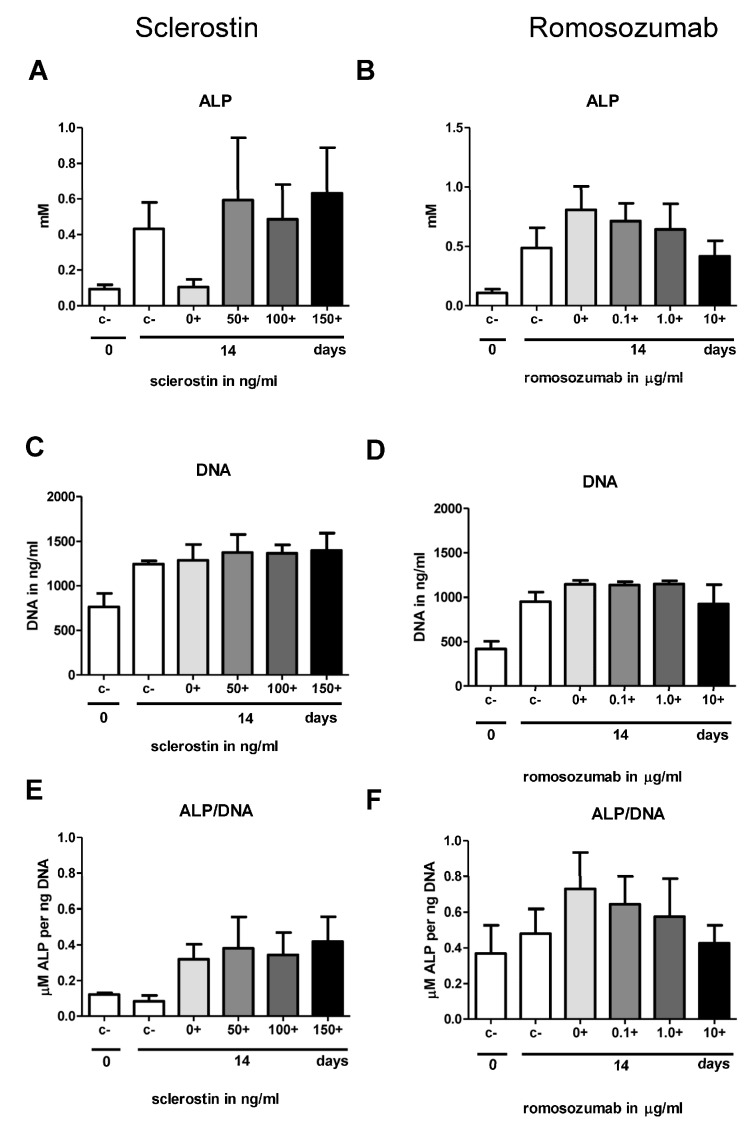
Alkaline phosphatase at t = 0 and t = 14 days. Alkaline Phosphatase (ALP) (**A**,**B**); DNA (**C**,**D**); Alkaline Phosphatase Activity (ALP/DNA) (**E**,**F**). (**A**,**B**) No statistical differences were observed in ALP between the different concentrations of both sclerostin and romosozumab. (**C**) Average DNA with increasing concentrations of sclerostin. (**D**) Average DNA with increasing concentrations of romosozumab. (**E**) ALP/DNA is slightly, but not significantly, increased with higher concentrations of sclerostin. (**F**) ALP/DNA appears to decrease slowly. No significant differences were observed in ALP/DNA between the different concentrations of romosozumab. Control c−conditions (days 0 and 14) were created without (−) mineralization medium and with mineralization medium (+) and with different concentrations of sclerostin (0+ to 150+) and romosozumab (0+ to 10+). Assays were performed using three (sclerostin) or five (romosozumab) different donors in duplicates. Average results ± SEM are shown.

At day 0 and day 14, ALP activity and DNA content were measured. Increasing concentrations of sclerostin or romosozumab were added to the mineralization medium. With increasing concentrations of sclerostin, ALP seems to increase slightly (Figure 3A). With increasing concentrations of romosozumab, ALP seems to decrease (Figure 3B). No statistical difference between the different conditions for both sclerostin and romosozumab were found. Large variations in ALP were observed between the different donors per concentration. The same samples were analyzed for DNA content. The number of cells had increased by day 14 compared to day 0 (Figure 3C,D). On day 14, the proliferation of cells was similar for different concentrations of sclerostin and romosozumab. Little variation was visible in DNA content between the different conditions. The addition of sclerostin or romosozumab did not affect the proliferation of PDL fibroblasts. The calculated ALP/DNA, or the ALP corrected for the number of cells, appears to have increased for increasing concentrations of sclerostin (Figure 3E) and appears to have decreased for increasing concentrations of romosozumab (Figure 3F). However, the calculated ALP/DNA was not significantly different for both sclerostin and romosozumab. 

#### 2.2.3. Alizarin Red Staining

To assess the mineral deposition of the PDL fibroblasts, alizarin red staining was conducted at day 21 for sclerostin (donors 1 to 3) and at day 14 and 21 for romosozumab (donors 4 to 8). Since we hypothesized that romosozumab would increase mineral deposition, we considered it appropriate to include two time points.

**Figure 4:**
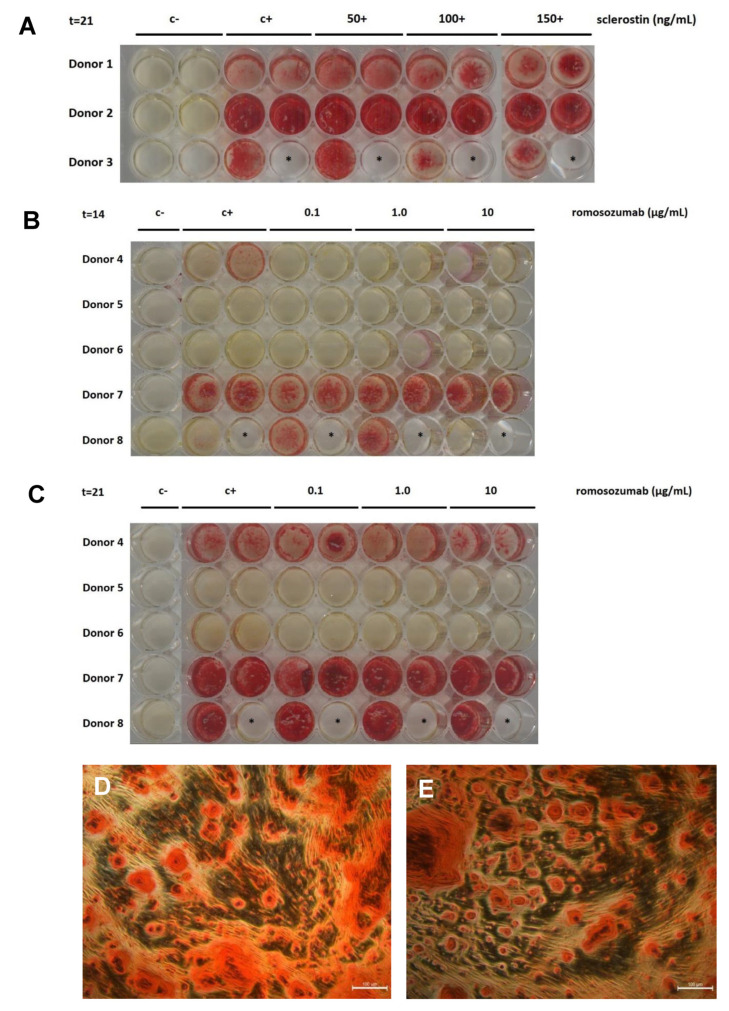
Alizarin red staining after sclerostin or romosozumab treatment. (**A**) PDL fibroblasts were cultured without (c−) or with (0+) mineralization medium under different concentrations of sclerostin (ng/mL) at t = 21 (**A**) or romosozumab at t = 14 (**B**) and t = 21 (**C**). Strong heterogeneity was observed between the donors. (**D**,**E**) Micrograph of stained PDL fibroblasts. Formation of red nodules is visible under the microscope: donor 1 with addition of 50 ng/mL sclerostin (**D**); donor 1 with addition of 100 ng/mL sclerostin (**E**). Red nodules with black speckles indicate mineral deposition. Scale bar represents 100 µm. *: empty wells.

Alizarin red staining showed substantial heterogeneity between the different donors for both sclerostin and romosozumab (Figure 4A–C). When cultured with the mineralization medium, PDL fibroblasts can be induced to form mineralized nodules (Figure 4D,E). For both experiments, calcium-rich deposits were only visible after the mineralization medium was added (Figure 4A–C). For donors 1 and 2, mineralization was comparable for different concentrations of sclerostin. Noticeably, mineralization nodules of donors 1, 2, and 3 seemed to be more concentrated in the center of the well with higher concentrations of sclerostin. For donor 3, there appeared to be less mineralization with the addition of 100 or 150 ng/mL sclerostin in comparison to the other conditions.

For the donor cells that were cultured with romosozumab, alizarin red staining was not only performed at day 21 but also at the earlier time point at day 14 since we hypothesized that romosozumab would initiate bone mineral deposition. In general, more mineralization was visible after 21 days compared to after 14 days. 

For donor 4, mineralization was only observed after 14 days in the control group and was very limited in the 10 µg/mL romosozumab group. After 21 days, mineralization was present at all concentrations of romosozumab with no difference in the level of mineralization between the different concentrations. No mineralization was observed for donor 5 after 14 days nor after 21 days. For donor 6, no mineralization at day 14 nor day 21 was observed either, with the exception of the group with the concentration of 1.0 µg/mL romosozumab. Donor 7 showed strong mineralization at the different concentrations for both day 14 and day 21. However, no differences in the level of mineralization were found between conditions per time point. Donor 8 showed an increased level of mineralization at 14 days with increasing levels between 0.1 and 1.0 µg/mL of romosozumab. However, mineralization was absent at 10 µg/mL romosozumab in this donor. After 21 days, for donor 8, strong mineralization was visible for all concentrations. The mineralization and the formation of red nodules was confirmed with micrographs (Figure 4D,E). 

In summary, the level of mineralization is generally not affected by the addition of sclerostin or romosozumab. This indicates that mineral deposition by PDL fibroblasts is not influenced by the addition of high concentrations of sclerostin or romosozumab. 

#### 2.2.4. The Influence of Romosozumab on Osteogenic Markers

**Figure 5:**
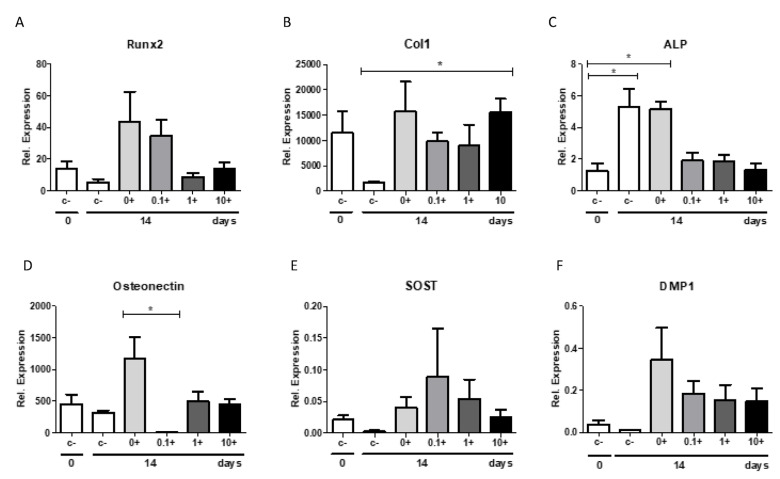
Relative expression of osteogenic markers in a PDL culture without (−) or with (+) mineralization medium under increasing concentrations of romosozumab. Cells were cultured for 14 days in the absence or presence of romosozumab at 0.1, 1.0, and 10 µg/mL. An amount of 10 µg/mL HI romosozumab was added to the control group with mineralization (0+). RNA was extracted and qPCR was performed to detect the relative expression of (**A**) RunX2, (**B**) Col1, (**C**) ALP, (**D**) Osteonectin, (**E**) SOST, and (**F**) DMP1. (**A**) RUNX2 shows no significant differences between different conditions. (**B**) Col1 shows a significant difference to untreated control (t = 14) for romosozumab at 10 µg/mL. (**C**) ALP was significantly elevated at t = 14 in the control cultures with and without mineralization in comparison to t = 0. (**D**) Osteonectin reduced significantly in the 0.1 µm/mL group compared to the control group. (**E**) SOST showed no significant differences between different conditions. (**F**) Relative expression of DMP1 was comparable between all conditions. Mean results ± SEM of 5 donors are presented. * *p* < 0.05.

Since we primarily expected a beneficial effect of romosozumab on osteogenic markers, we used qPCR to assess the effects on these markers only in cultures with romosozumab. The effect of romosozumab on the expression of the different osteogenic markers, RUNX2, Osteonectin, and Col1, was assessed in the PDL cultures at day 14 (Figure 5). 

The early osteogenic marker, transcription factor RUNX2, seemed to be higher, but not significantly higher, at day 14 after the addition of the mineralization medium in comparison to day 0. No significant differences were found between the different conditions or over time for RUNX2. 

Collagen type I was assessed as a marker for matrix deposition. Col1 was expressed more highly after adding the mineralization medium. The expression of Col1 on day 14 in the control group without the mineralization medium was significantly decreased in comparison to that of the same timepoint in the group with the addition of 10 µg/mL romosozumab. For Col1 between different romosozumab conditions, no significant differences were observed. 

Alkaline phosphatase, ALP, an enzyme associated with calcium deposition and an intermediate marker, was significantly upregulated at day 14 in the control cultures with and without mineralization in comparison to day 0. Noticeably, the mineralization medium appears to have had little influence on ALP expression. No difference in the relative expression of ALP with and without the mineralization medium was detected within the control group. Although not significantly, the addition of romosozumab seems to decrease the relative expression of ALP.

The late osteogenic marker, osteonectin, a bone matrix specific protein, was upregulated at day 14 with the addition of the mineralization medium. The osteonectin gene expression reduced significantly in the 0.1 µg/mL romosozumab group compared to in the control group. Between the other concentrations and the control group, no significant difference was identified. 

The relative expression of sclerostin of SOST and DMP1 was relatively low. The low relative expression of DMP1 is probably explained by the fact that DMP1 is mainly produced in osteocytes, which were absent in the present study. For SOST, no significant differences were observed between the control conditions and the different concentrations of romosozumab. It is likely that SOST is produced less by PDL fibroblasts than hypothesized. 

The effect of the mineralization medium is clearly visible for DMP1. After adding this medium, the relative expression of DMP1 is strongly enhanced. However, no significant differences were found between different concentrations of romosozumab. 

In general, the qPCR analysis indicated that PDL fibroblasts are only slightly affected by the addition of romosozumab. In addition, this analysis shows that the addition of the mineralization medium increases the expression of the studied osteogenic markers. 

#### 2.2.5. PDL Fibroblasts Express Low Levels of SOST and LRP-4 and LRP-5 Compared to Bone-Embedded Osteocytes

**Figure 6:**
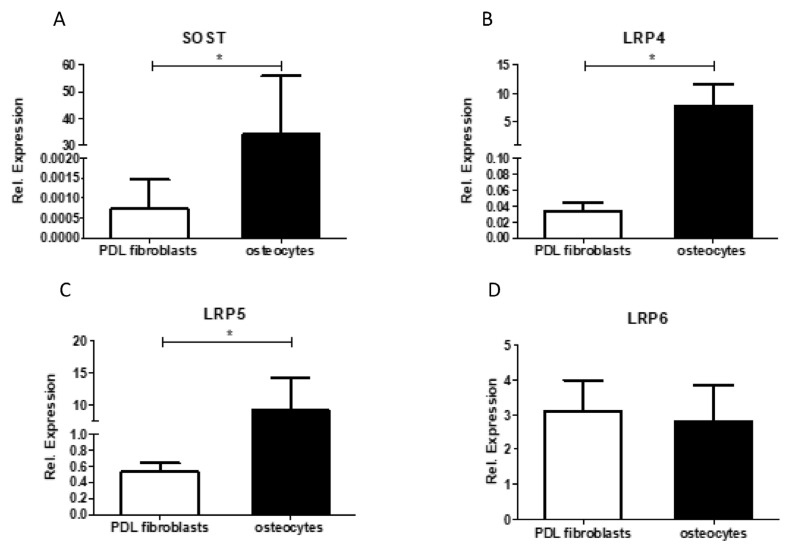
Relative expression of sclerostin and sclerostin receptors LRP 4, 5, and 6 in PDL fibroblasts (n = 5) and in osteocytes (n = 4) from bone isolates. (**A**) Sclerostin or SOST mRNA expression was significantly higher than that of PDL fibroblasts and 45,000 times the expression of osteocytes. The relative expression of (**B**) LRP 4 (450×) and (**C**) LRP 5 (17×) in PDL fibroblasts was significantly lower in comparison to the relative expression of LRP 4 and 5 in osteocytes. (**D**) Relative mRNA expression of LRP 6 in PDL fibroblasts and osteocytes was comparable. Mean results ± SEM are presented. Significant results are shown with a black bar (* *p* < 0.05). A Mann–Whitney U test was used.

Finally, to account for the apparent lack of response to sclerostin or romosozumab, we compared the gene expression of sclerostin or SOST and its receptors LRP-4–6 to genuine producers of these proteins (Figure 6). Five cultures of PDL were compared with four isolates of mRNA from osteocyte-rich bone. Expressions of SOST (average expression PDLs 0.001 vs. 34 in osteocytes), LRP-4 (PDLs 0.03 vs. osteocytes 7.7), and LRP-5 (0.5 vs. 9.3) were significantly higher in mRNA extracted from bone-containing osteocytes. The only exception was LRP-6 (3.1 vs. 2.8), in which comparable levels were measured between PDLs and osteocytes. The enormous differences in the expression between cultured PDL fibroblasts and those isolated from osteocytes could explain the apparent lack of response to sclerostin or romosozumab. 

## 3. Discussion

The present research was the first to assess the effects of both sclerostin antibodies and a monoclonal sclerostin antibody (romosozumab) on osteoclastogenesis and osteogenesis in PDL cultures. The PDL fibroblasts uniquely contribute to both osteoclastogenesis and osteogenesis [28] and, therefore, both processes could be studied in the same experimental approach. Although both reagents were added until high dosages, there were limited effects on both osteogenesis and osteoclastogenesis. The low expression of sclerostin and its receptors could be the cause for this. 

Regarding osteoclastogenesis, no studies have previously found that sclerostin could influence osteoclast formation in PDL-PBMC co-cultures. Here, our results suggest that the addition of a high concentration of romosozumab slightly decreases the osteoclastogenic capacity of PDL-PBMC co-cultures. 

In this study, we found no effect of sclerostin on osteoclast formation in a co-culture of PDL fibroblasts and PBMCs. A possible explanation could be that osteocytes, in particular, could be receptive to sclerostin and, hence, influence osteoclast formation. Osteocytes have previously been shown to be key expressors of RANKL [29]. It has been reported that sclerostin affects osteoclasts by upregulating the RANKL/OPG ratio [30]. Osteoclast precursor cells express receptor RANK on their cell surface [31]. This receptor is able to bind to its ligand RANKL, which is highly expressed by osteocytes [31]. Binding RANK to RANKL stimulates osteoclast precursors to differentiate into multinucleated osteoclasts [31]. Osteocytes also produce osteoprotegerin (OPG), which inhibit osteoclast differentiation by competing with RANKL for its receptor RANK on osteoclast precursor cells [32]. In addition to osteocytes, PDL fibroblasts also express RANKL and OPG mRNA [28,32], although the expression of OPG is at least 100-fold higher than RANKL [28]. It was hypothesized that sclerostin might stimulate osteoclast formation indirectly via PDL by stimulating RANKL expression. However, it could also be that sclerostin only affects osteoclast formation indirectly via RANKL produced by osteocytes and not in PDL fibroblasts, which may express lower levels of receptors for sclerostin. This thought is consistent with the findings of Wijanayaka et al., who showed that sclerostin dose-dependently upregulated the expression of RANKL mRNA and downregulated OPG mRNA in a PBMC-osteocyte co-culture [33]. However, this idea is not supported by the research of Odagaki et al. [34]. They found no effect in RANKL or OPG expression in osteocytes in their PDL-osteocyte co-culture system and they attributed an increased bone resorption to the RANKL/OPG signaling of the PDL fibroblasts [34]. 

This study also showed that romosozumab causes a slight but significant decrease in the formation of TRAcP-positive cells at high concentrations of romosozumab. Previous research showed that sclerostin induces the RANKL/OPG ratio and romosozumab reduces this ratio [35]. The current study showed that romosozumab decreases the number of TRAcP-positive cells, indicating that osteoclastogenesis is inhibited, possibly by decreasing the RANKL/OPG ratio of PDL fibroblasts. Thus, this result is in line with romosozumab’s alleged effect on the RANKL/OPG ratio, which is also suggested by Odagaki et al. [34], albeit this ratio is below 1/100 in most studies analyzed by Sokos et al. [28]. Future research should focus on which cell type(s) sclerostin influences RANKL/OPG expression. The lack or low expression of sclerostin receptors LRP-4 and LRP-5, as shown in Figure 6, make it unlikely that added or endogenous sclerostin will influence RANKL-expression in PDL fibroblasts. 

Moreover, this study also revealed that neither sclerostin nor romosozumab influenced the spontaneous osteoclast formation of PBMCs. These results substantiate the hypothesis that sclerostin depends on other cell types to have an effect on osteoclast formation. In addition, thus far, it has not been reported that PBMCs express either sclerostin or its receptors [36]. These results are in line with the data from Wijenayaka et al., who also found no effect of sclerostin on TRAcP-positive cell formation in the monocultures of PBMCs, albeit these cultures were supplemented with macrophage colony-forming factor M-CSF [33]. 

It is important to note that, in the current study, osteoclastogenesis was determined according to the number of TRAcP-positive multinucleated cells. We have previously shown that PDL fibroblasts do provide the signals for the generation of multinucleated TRAcP-positive cells but that this does not lead to resorbing osteoclasts, for which extra RANKL is needed [27]. It should be acknowledged that the resorptive activity of osteoclasts was, therefore, out of scope. Wijenayaka et al. reported that in a co-culture of PBMCs and osteocytes (MLO-Y4 cells) sclerostin can specifically result in increased resorbing activity without increasing the number of TRAcP-positive cells [33]. 

In the second part of this study, we investigated osteogenesis, the biological process most widely studied in the context of sclerostin. Contrary to expectations, this study showed that neither sclerostin nor romosozumab affected the osteogenic potential of the PDL fibroblasts. Firstly, the addition of both sclerostin and romosozumab did not show any significant increase or decrease in ALP activity. Secondly, mineral deposition by PDL fibroblasts did not seem to be affected by the addition of higher sclerostin or romosozumab levels. The present study did not show any significant increase or decrease in ALP activity by the addition of both sclerostin and romosozumab. Bezooijen et al. studied the effect of osteoblastic (KS483) cell cultures with added sclerostin [37]. They observed that sclerostin inhibited APL activity only at the highest concentration of 2.5 μg/mL [37]. They suggest that sclerostin only inhibits the later stages of bone formation and, therefore, has no effect on an early osteoblast differentiation marker such as ALP activity [37]. This suggestion could also apply to the results of the present study. 

For osteogenesis assays, cells were seeded and cultured in the mineralization medium with β-glycerol phosphate and ascorbic acid to stimulate osteogenic differentiation. No staining was observed in cultures with the control medium (without β-glycerol phosphate and ascorbic acid). In contrast, deposits of alizarin red were observed in cultures that contained the mineralization medium from day 14. At day 21, mineralization was visualized with alizarin red staining for six out of eight donors. These results reflect those of Knaup et al., who also found mineralization by PDL fibroblasts under mineralizing conditions from day 13 [11]. Since we hypothesized that romosozumab would initiate bone mineral deposition, alizarin red staining was performed not only at day 21 but also at day 14. However, an initiation of bone mineral deposition with increasing levels of romosozumab could not be determined. Although only a qualitative analysis was performed, no differences were observed for any concentration of sclerostin or romosozumab. A more quantitative approach, by dissolving the red stain, could have enforced these findings. Moreover, it was noticed that there was a great level of heterogeneity in the level of mineralization between the different donors. This finding aligns with Knaup et al., who demonstrated differences between donor cells regarding their mineralization potential to form mineralized deposits of alizarin red and subsequent osteogenic differentiation potentials [11].

In the present study, a qPCR with different osteoblast-related genes was performed. When comparing the relative expression with and without the mineralization medium, all studied bone markers showed a strong increase in relative expression after the addition of the mineralization medium, resulting in a high anabolic potential of the PDL fibroblasts. Romosozumab did not induce the expression of the following bone-formation markers: ALP, osteonectin, Runx2, Col1, and DMP1.

It was shown in the first report of PDL cell cultures [38], and shown in subsequent studies [24,25,27], that the cultures vary largely, especially in the expression of alkaline phosphatase and in mineralization, as assessed by alizarin red. In the present study, a large variation in these parameters was also observed, as shown in the alizarin red staining in Figure 4B,C, the alkaline phosphatase staining in Figure 3A,B, and the sclerostin expression in Figure 6A. Despite this variation, the results could be analyzed by repeated measures: each donor was exposed to the varying concentrations of sclerostin or romosozumab.

It should be acknowledged that the relative expression of SOST mRNA was relatively low in this study in comparison to the other osteoblastic markers. The SOST mRNA expression was between 70,000-fold and 633,000-fold lower than the expression of Col1, between 148-fold and 1333-fold lower than the expression of RUNX2, between 30-fold and 60,000-fold lower than the expression of osteonectin, and between 15-fold and 1083-fold lower than the expression of ALP. The low level of SOST mRNA in the cell cultures suggests that SOST mRNA is primarily restricted to a few cell types, and most likely osteocytes. Indeed, we also confirmed in the present study that the expression of SOST and LRP4 and LRP5 was much higher in osteocytes than in PDL fibroblasts. Similarly, in previous research, Bezooijen et al. suggested that SOST mRNA expression might be restricted to osteocytes [37]. 

Another possible explanation for the low relative expression of SOST mRNA may be the differentiation-dependent expression of SOST mRNA, as also suggested by Jäger et al. [12]. Bezooijen et al. investigated the onset of SOST mRNA expression in cultured human and mouse MSCs and KS483 cells under osteogenic conditions by adding the mineralization medium [37]. They reported that SOST mRNA expression could not be detected in undifferentiated cells but was always detectable in late differentiated cells [37]. Furthermore, SOST mRNA expression was limited to the mineralization phase of osteoblastic cell cultures [37]. Thus, they concluded that the onset of SOST mRNA depends on the differentiation stage of the cells. In addition, some researchers indicate that PDL fibroblasts grown in cultures represent an immature form of PDL fibroblasts [39]. The possible immature form of the PDL fibroblasts in the studied cultures could explain why low SOST mRNA expression was found. 

Alternatively, it could be that PDL fibroblasts hardly express SOST at the protein level; hence, romosozumab would not have any effect on the expression of the studied bone formation markers. Similarly, the absence of an effect of sclerostin could be due to the relatively low expression of receptors for sclerostin (LRP4, 5, or 6) in PDL fibroblasts, resulting in an absent SOST signaling. 

In line with the low expression of SOST mRNA was the low expression of DMP1, which is also typically associated with osteocyte expression. DMP1 is known as an osteocyte-specific gene, as it plays an important role in osteocyte maturation [31]. Thus, the results indicate that differentiation as induced by osteogenic conditions did not lead to differentiation in the osteocyte-like phenotype.

The limited effect of both sclerostin and romosozumab on osteogenesis and osteoclastogenesis observed in the current study might be caused by the function of the PDL itself. An important biological function of PDL fibroblasts is the maintenance of PDL homeostasis by not only preventing osteoclast formation at the surface of the root [28] but also preventing too much bone formation by releasing locally acting regulators to avoid tooth ankylosis [24]. This protective function of the PDL may allow little or no interference of a negative bone regulator of bone formation such as sclerostin. In this context, Knaup et al. assumed that sclerostin might be part of a negative feedback loop in PDL fibroblasts in which an enhanced level of sclerostin could inhibit mineralization formation during osteogenic differentiation to prevent extreme mineralization [11]. They examined that PDL fibroblasts with low baseline SOST levels demonstrate earlier mineralization than those with higher initial SOST levels [11]. Contrarily, high levels of SOST resulted in a smaller decrease in the formation of mineralization by PDL fibroblasts and decreased osteogenic differentiation [11]. In addition, Manokawinchoke et al. suggested a role for sclerostin in PDL homeostasis [22]. They proposed that mechanical forces induce the expression of sclerostin and thereby prevent bone deposition in the PDL space [22].

One of the limitations of this study is the heterogeneity between the different PDL fibroblast cultures from the different donors. Marchesan et al. recommended a large donor base for PDL cultures to normalize for cellular heterogeneity and various factors that can influence the cultured cells [40]. 

Moreover, in the current study, romosozumab only showed a small effect on the expression of osteogenic markers. Sclerostin reduces osteoblastic bone formation by antagonizing the canonical Wnt/β-catenin signaling pathway by binding with the Wnt co-receptors, LRP 5/6 [7]. LRP 4 was also discovered as a sclerostin receptor [8]. Furthermore, sclerostin also has inhibitory effects on the bone morphogenetic protein signaling pathway [41]. In addition to these two pathways, other pathways have been suggested [42,43]. Research should show which receptors for sclerostin contribute to the effects seen in this study and on which pathway(s) these effects depend. Further studies should include LRP receptors, as research suggests that LRP5 depletion in mice affects the structure of the PDL fibroblasts and reduces osteoclastogenesis activity in the PDL [44]. 

In conclusion, this study showed that both sclerostin and romosozumab, individually, had very limited effects on osteoclastogenesis and osteogenesis in PDL fibroblast cultures. PDL fibroblasts likely do not express enough sclerostin or sclerostin receptors to induce any effect. The limited interaction of sclerostin with PDL fibroblasts may be explained by the intrinsic properties of the PDL fibroblasts themselves, as they must resist excessive bone formation and bone degradation under physiological circumstances.

## 4. Materials and Methods

### 4.1. Sclerostin and Monoclonal Sclerostin Antibody

Recombinant human sclerostin was acquired from R&D Systems (Minneapolis, MN, USA) and dissolved in water. Biological mode of action was tested at R&D in a cell line, MC3T3-E1, that responds to Wnt-3a, in which sclerostin-inhibited alkaline phosphatase activity in the range of concentrations that we have used takes place. Monoclonal sclerostin antibody (Amgen^®^, Thousand Oaks, CA, USA, romosozumab, EU/1/19/1411/001, CNK: 4230-116) was purchased as Evinity (trademark), hereinafter referred to as romosozumab. It was collected from one of the pens that are used for treating osteoporosis patients. All romosozumab for clinical use was subjected to stringent quality control. Romosozumab was further diluted 9 times in water, with a stock solution of 10 mg/mL, which was further diluted 1000× for the highest concentration used in the assays. 

As an additional control, containing the same solvent, monoclonal sclerostin antibody (HI romosozumab) was inactivated by heating the romosozumab for 10 min at 90 °C. HI romosozumab was used as a control in some of the experiments. 

### 4.2. Isolation and Culturing of PDL Fibroblasts

PDL fibroblasts were isolated from roots of the third molars of eight healthy individuals between 18 and 25 years of age. All individuals underwent extraction of the third molar at Amsterdam UMC (location VUmc, Amsterdam, The Netherlands), no inflammation was visible at the site of extraction. All samples were retrieved with written informed consent and approved by the medical ethical committee of Vumc (2016.105) and ACTA-ETC (2021-55908). No signs of caries, gingivitis, or periodontitis were present (pockets ≤ 3 mm without bleeding). For all experiments, researchers were not aware of the identity of the PDL donors. Cells were stored in liquid nitrogen. PDL was harvested by scraping the cells off the third molar. Additionally, cells were seeded in a culture medium consisting of 90% Dulbecco’s minimal essential medium (DMEM, Thermo Fischer Scientific, Waltham, MA, USA), 10% fetal clone I (FCI, HyClone, Logan, UT, USA), and 1% antibiotics (100 U/mL penicillin, 100 µg/mL streptomycin, and 250 ng/mL Amphotericin B; Sigma-Aldrich, St. Louis, MO, USA) and maintained in a humified atmosphere of 5% CO_2_ in air at 37 °C. All assays were conducted with 4th or 5th passage cells.

### 4.3. Isolation of Osteocyte Enriched mRNA

Fibular bone was collected from 4 donors (age: 32–79, 1 female, 3 male). The donors presented no medical history of skeletal pathology or trauma. All bone tissue was collected as surgical waste during mandible reconstruction surgery and obtained with donors’ consent. All protocols were approved by the local Medical Ethical Committee of the Amsterdam University Medical Centers (2016.105). Fibular bone explants were washed in Hanks’ balanced salt solution (HBSS; Thermo Fisher Scientific, Waltham, MA, USA). They were incubated in minimal essential medium (MEM; Thermo Fisher Scientific) containing 2 mg/mL type II collagenase (Worthington Biochemical, Lakewood, CA, USA) for 2 h in a shaking water bath at 37 °C and washed again twice with HBSS. Then, soft tissue was removed by scraping. Cleaned cortical bone explants were cut into small explants measuring 8.0 × 3.0 × 1.5 mm (l × w × h) using a diamond disc H-345-220 (Horico, Berlin, Germany), a handpiece (KaVo, Biberach an der Riss, Germany), and a foot control (KaVo), as described earlier [45]. Explants were cooled during cutting in ice-cold HBSS. They were pre-cultured for 1 or 2 days in 6-well plates (Merck KGaA, Darmstadt, Germany) containing MEM supplemented with 5% fetal bovine serum (FBS; Lonza BioWhittaker, Basel, Switzerland), 5% bovine calf serum (BCS; Thermo Fisher Scientific), 1% penicillin-streptomycin (10,000 U/mL; Thermo Fisher Scientific), and 0.5% amphotericin B solution (Merck KGaA, Darmstadt, Germany) at 37 °C. 

Bone explants were pulverized using a 6775 Freezer/Mill cryogenic grinder (SPEX SamplePrep, Metuchen, NJ, USA; rate: 10 cycles/s; 20 impacts/s) for 2 min in liquid nitrogen. After pulverization, the TRIzol/bone powder mixture was incubated for 1 h in a shaking water bath at 37 °C. Immediately thereafter, RNA was either isolated, or the TRIzol/bone powder mixture was stored at −80 °C before RNA isolation. Two hundred microliter chloroform was added per ml of TRIzol/bone powder mixture, followed by 15 min of centrifugation at 12,000 rpm at 4 °C. Isolation and purification of RNA was performed using RNeasy Midi columns (Qiagen, Hilden, Germany) or Zymo-Spin IIICG columns (Direct-zol RNA MiniPrep Plus, Zymo Research, Irvine, CA, USA). The supernatants were collected, mixed with an equal volume of 70% ethanol, and transferred to RNeasy Midi columns or were mixed with an equal volume of 100% ethanol and transferred to Zymo-Spin IIICG columns. RNA isolation and DNase I digestion were performed following the manufacturer’s instructions. The RNA concentration was determined by Nanodrop spectrophotometer (Nanodrop Technologies, Wilmington, DE, USA) and Qubit RNA HS assay kits (Thermo Fisher Scientific). 

### 4.4. Isolation of PBMCs

PBMCs from a buffy coat (Sanquin, Amsterdam, The Netherlands) of a healthy donor were isolated. Firstly, the buffy coat was diluted 1:1 with 1% PBS-citrate. The diluted buffy coat was carefully layered on Lymphoprep (Axisshield Po CAS, Oslo, Norway) and centrifuged for 30 min at 800 G without brakes. Next, the interphase with PBMCs was taken off, washed with 1% PBS-Citrate solution, and centrifuged for 10 min at 400 G. This was repeated three times. Finally, the supernatant was removed, and the pellet was resuspended in basal culture medium before culturing. Cells were counted with Muse^®^ Cell Analyzer. 

### 4.5. Osteoclastogenesis Assays

Different osteoclastogenesis assays were performed. A high density (HD) monoculture was plated with 0.5 × 10^6^ PBMCs four times in 96-well plates, cultured in DMEM, and supplemented with 10% FCI, 1% PSF, and an antibiotics cocktail containing penicillium, streptomycin, and fungisone. Sclerostin was added in concentrations of 50, 100, and 150 ng/mL. These relatively high concentrations were chosen based on a previous experiment (not described here) in which concentrations up to 50 ng/mL did not result in a significant effect.

For the co-culture, PDL at a density of 1.5 × 10^4^ cells per well were seeded in duplicate in a 48-well plate. The next day, 0.5 × 10^6^ PBMCs were added to each well. The test setups were made with the following co-culture: (1) Culture medium with solvent of sclerostin (water). (2) Culture medium + 50 ng/mL sclerostin. (3) Culture medium + 100 ng/mL sclerostin. (4) Culture medium + 150 ng/mL sclerostin at t = 0. The same experiments were performed with romosozumab with concentrations up to 10 µg/mL (in excess of 100,000 of the literature outcomes). For co-culture experiments, PDL were co-cultured with PBMCs in the presence of culture medium with (1) 10 µg/mL heat-inactivated romosozumab as a control; (2) 0.1 µg/mL romosozumab; (3) 1 µg/mL romosozumab; and (4) 10 µg/mL romosozumab.

Culture medium of all osteoclastogenesis assays was refreshed every 3 or 4 days. All plates were incubated at 37 °C and 5% CO_2_. Cells of osteoclastogenesis assays were harvested at t = 0 and t = 21 for tartrate-acid phosphatase (TRAcP) staining.

### 4.6. TRAcP Staining

After 21 days of culturing, cells of the osteoclastogenesis assays were washed with PBS, fixed in 4% formaldehyde for 10 min, and washed again with PBS. In accordance with the protocol of the manufacturer Sigma-Aldrich, the TRAcP staining solution was prepared. An amount of 300 µL of TRAcP staining solution was added to each well and the wells were incubated for 10 min in a dark place at room temperature. Then, nuclei were counterstained with 4′,6-diamidino-2-fenylindole (DAPI) for 5 min and washed with PBS. TRAcP-positive cells with three or more nuclei were labelled as osteoclasts. TRAcP-positive cells with three nuclei or more were counted using micrographs taken from a light microscope with a fluorescence device. Five (PBMCs HD culture) or three standardized (co-culture PDL-PBMC) areas were selected per well (magnification 20×) and photographed to determine the number of nuclei of TRAcP-positive multinucleated cells (MNCs). 

### 4.7. Osteogenesis Assays

#### 4.7.1. Sclerostin

PDL fibroblasts of three different donors were seeded in duplicate in 48-well plates (3.0 × 10^4^ cells/well). Mineralization medium was used to induce and stimulate mineralization by the PDL fibroblasts [12]. The following test conditions were used: (1) culture medium; (2) mineralization medium, comprising culture medium + 50 mM β-glycerophosphate (Sigma-Aldrich, St. Louis, MO, USA) + 50 mM ascorbic acid (Sigma-Aldrich); (3) mineralization medium + 50 ng/mL sclerostin; (4) mineralization medium + 100 ng/mL sclerostin; (5) mineralization medium + 150 ng/mL sclerostin. The concentrations of sclerostin were determined based on comparable previously conducted studies with osteocytes or PDL fibroblasts [33]. Cells were harvested for ALP activity assay at t = 0 and t = 14, and alizarin red staining was performed at t = 21. 

#### 4.7.2. Romosozumab

PDL fibroblasts of five different donors were seeded and cultured in the same way as described for sclerostin osteogenesis assay. Romosozumab was used in concentrations of 0.1, 1.0, and 10 µg/mL. HI romosozumab was added to the control group with mineralization medium. Cells were harvested for ALP activity assay at t = 0 and t = 14, alizarin red staining at t = 14 and t = 21, and qPCR analysis at t = 0 and t = 14 for the examination of osteogenic markers. 

Culture medium of all osteogenesis assays was replaced twice a week. All plates were incubated during the experiments at 37 °C and 5% CO_2_.

### 4.8. Alkaline Phosphatase Activity and DNA Concentration

At day 0 and day 14 cells from the osteogenic assays were harvested for measurements of alkaline phosphatase (ALP) activity and DNA concentration. Cells lysates were achieved by washing the cells first with PBS, adding 200 µL Milli-Q water to each well, and then freezing the cells at −20 °C. Subsequently, after three cycles of freeze-thaw, adherent cells were scraped off the well with a plastic cell scraper. ALP was determined using 4-nitrophenyl phosphate disodium salt at pH 10.3. After incubating the plate for one hour at 37 °C, the reaction process was blocked with NaOH. With a Synergy HT spectrophotometer (BioTek, Instruments, Inc. Winooski, VT, USA), absorbance was measured at 405 nm. 

Using the CyQuant Cell Proliferation Assay Kit (Molecular Probes (C7026), Leiden, The Netherlands), DNA concentrations were measured from the same cell lysates that were used for the ALP. For the calculation of DNA concentration, fluorescence was measured according to the protocol using the Synergy HT spectrophotometric microplate reader. 

### 4.9. Alizarin Red Staining

After 14 days and 21 days of culturing, mineral deposition was inspected in duplicate or single wells per donor. Cells were washed with PBS, fixed for 10 min in 4% PBS buffered formaldehyde and then washed with Milli-Q water. For the staining, 300 µL of 1% Alizarin Red staining solution at pH 4.3 (Sigma-Aldrich, St. Louis, MO, USA) was added to each well and cells were incubated for 10 min at room temperature and rinsed with Milli-Q water until the background was clear. Mineral deposition was visible via the formation of red precipitate, which was often in the form of nodules. 

### 4.10. Quantitative Polymerase Chain Reaction (qPCR)

At day 0 and day 14 RNA was isolated from cultured cells with different concentrations of romosozumab. Firstly, for adherent cells the supernatant was removed and lysis buffer containing β- Mercaptoethanol (150 µL t = 0 and 200 µL t = 14) was added. Samples were stored at −80 °C. The RNA from the different samples was extracted according to the manufacturer’s protocol of the RNeasy kit (Qiagen, Düsseldorf, Germany). Finally, RNA concentration was measured with a Synergy HT spectrophotometer. 

For the synthesis of first strand cDNA from the RNA samples, a Fermentas kit (First strand cDNA synthesis kit Fermentas #K1612) was used. For one reaction, 9 µL RNA was mixed together with 2 µL primer mix consisting of Oligo (dT18) and D(N)6 primers, incubated at 65 °C for 5 min, and then put on ice. An amount of 9 µL master mix, consisting of buffer (4 µL), dNTP’s (2 µL), RNAse Inhibitor (1 µL) and Reverse Transcriptase (2 µL) was added. The mix was successively incubated at 25 °C for 5 min, at 37 °C for 1 h, and at 70 °C for 5 min. cDNA was diluted to 1 ng/µL and samples were stored at −20 °C. First strand cDNA samples were used as a template in the qPCR. 

PCR primers of osteoblast-related genes were designed (Table 1). An amount of 1 µM of each PCR primer was added to the cDNA samples as described in the previous section. qPCR was performed on the LightCycler^®^ (Roche). qPCR started with an initial activation step for 10 min at 94 °C, followed by a run of 40 cycles. This cycle run comprised a template denaturation step at 94 °C for 30 s and the primer annealing and primer extension step combined at 60 °C for 1 min. 

Low expression housekeeping gene Porphobilinogen deaminase (PBGD), a gene without pseudogenes, was used to normalize the samples. The expression of PGBD was not influenced by experimental factors. By calculating ΔCT, samples were normalized for the expression of PGBD. Relative expression of the different genes is expressed (2^−(ΔCT)^). 

### 4.11. Statistical Analysis

For analyzing the data sets, Graphpad Prism 5 software was used. Comparisons between the different groups in the number of osteoclasts and the number of nuclei per cell were analyzed using one-way ANOVA followed by a Kruskal–Wallis post-test. For analyzing ALP activity, DNA levels, and relative expression of different osteogenic markers, a Friedman test was used, followed by Dunn’s multiple comparison test. All the data are shown as mean ± SEM. *p* values of *p* < 0.05 were considered to indicate statistical significance. Mineral deposition by PDL fibroblasts (alizarin red staining) was detected visually. All statistical tests were repeated measurement tests, where n = 3 or n = 5 were used for comparison per donor.

## Figures and Tables

**Table 1:** Primer sequences used for quantitative polymerase chain reaction (qPCR).

Gene	Sequence	Amplicon Length	Ensembl Gene ID
Osteogenesis			
DMP1	5′ CCTCTTTGAGAACATCAACCTGATTT 3′	106	ENSG00000152592
	5′ GAGCAGGATGCTGATCTTCATAGTT 3′		
RUNX2	5′ ATGCTTCATCGCCTCAC 3′	156	ENSG00000124813
	5′ ACTGCTTGCAGCCTTAAAT 3′		
COL1A1	5′ TCCAACGAGATCGAGATCC 3′	190	ENSG00000108821
	5′ AAGCCGAATTCCTGGTCT 3′		
Osteonectin	5′ TACATCGGGCCTTGCAAATAC 3′	100	ENST00000231061
	5′ AGGGTGACCAGGACGTTCTTG 3′		
ALP	5′ GCTTCAAACCGAGATACAAGCA 3′	101	ENSG00000162551
	5′ GCTCGAAGAGACCCAATAGGTAGT 3′		
Sclerostin/ SOST	5′ GGGTGGCAGGCGTTCA 3′	163	ENSG00000167941
	5′ CTGTACTCGGACACGTCTTTGGT 3′		
LRP-4	5′CAAGGAGTTCCGCTGTAGTGA 3′	187	ENSG00000134569
	5′CGCCATCGCAGTGGTAGATGT 3′		
LRP-5	5′ GCATGGCCGTTGACTGGAT 3′	175	ENSG00000162337
	5′ CCACTCGGTCCAGTAGATGTA 3′		
LRP-6	5′ CAGCACCACAGGCCACCAA 3′	226	ENSG00000070018
	5′ TCGAGACATTCCTGGAAGAG 3′		
PBGD	5′ TGCAGTTTGAAATCATTGCTATGTC 3′	84	ENSG00000113721
	5′AACAGGCTTTTCTCTCCAATCTTAGA 3′		

## Data Availability

Data from this project are available upon request.
